# Does mRNA-based COVID-19 vaccination in the subacute phase lead to microstructural brain changes? A prospective pilot MRI study using T1 relaxometry

**DOI:** 10.1038/s41598-026-56181-6

**Published:** 2026-06-11

**Authors:** Rafael Willems, Marta Cappelletti, Jan R. Schüre, Ralf Deichmann, Elke Hattingen, Christophe T. Arendt

**Affiliations:** 1 Institute of Neuroradiology, Goethe University Frankfurt, University Hospital, Theodor-Stern-Kai 7, 60596 Frankfurt, Germany; 2 Cooperative Brain Imaging Center - CoBIC, Goethe University Frankfurt, Frankfurt, Germany

**Keywords:** Quantitative MRI, Brain inflammation, Magnetic resonance imaging, mRNA vaccines, Severe acute respiratory syndrome coronavirus 2, Diseases, Medical research, Neurology, Neuroscience

## Abstract

Messenger ribonucleic acid (mRNA)-based vaccines delivered via lipid nanoparticles (LNP) were pivotal in managing the severe acute respiratory syndrome coronavirus (SARS-CoV-2) pandemic. While mild systemic reactions are common after vaccination, rare neurological complications have raised concerns about potential structural cerebral changes. This prospective pilot study tested the hypothesis that LNP-mRNA-based SARS-CoV-2 vaccination does not induce significant microstructural cerebral changes in healthy adults. Dedicated 3 Tesla brain MRI was performed at three time points: up to one month before the first vaccination (t1), and 1–2 weeks (t2) and 2–3 months (t3) after the second dose of either BNT162b2 or mRNA-1273. Across 85 MRI scans from 29 initially SARS-CoV-2-seronegative adults, 2D T2-weighted fluid-attenuated inversion recovery, synthetic 3D T1 magnetization prepared rapid acquisition of gradient echoes and 3D quantitative T1 mapping were analyzed. Changes in w matter hyperintensities (WMH) were evaluated visually (Prins scale) and semiquantitatively (Longitudinal Brain Imaging). Microstructural changes of deep and cortical gray matter and white matter were analyzed using qT1 values via paired t-tests or Wilcoxon signed-rank tests. No new or progressive WMH nor significant intraindividual qT1 changes were observed. QT1% deviations were small (mean 0.1%, SD 0.9%; maximum 2.08%) and remained within established scan-rescan variability limits at 3 T. Apart from a 1 mm decrease of a preexisting WMH in one participant, no visually detectable structural brain alterations were observed. Our findings provide no evidence for WMH progression or microstructural cerebral changes after LNP-mRNA-based SARS-CoV-2 vaccination in healthy adults in the subacute phase.

## Introduction

Coronavirus disease 2019 (COVID-19), caused by the severe acute respiratory syndrome coronavirus 2 (SARS-CoV-2), led to a global pandemic. While primarily affecting the respiratory system, COVID-19 is also considered a multisystemic disease involving the nervous system^1–3^. In response, messenger ribonucleic acid (mRNA)-based vaccines such as BNT162b2 (Comirnaty^®^, Pfizer and BioNTech, New York, NY/USA and Mainz, Germany) and mRNA-1273 (Spikevax^®^, Moderna, Inc., Cambridge, MA/USA) were rapidly developed and widely deployed^3^. For basic immunization, these vaccines are administered intramuscularly in two doses: 30 µg per dose, 21 days apart for BNT162b2, and 100 µg per dose, 28 days apart for mRNA-1273^5^.

To ensure stability and prevent degradation, the mRNA is encapsulated in lipid nanoparticles (LNP). After injection, LNP deliver mRNA to myocytes and immune cells, where the spike glycoprotein (S) is expressed to stimulate the immune response^6^. Although the S mainly remains at the injection site, small quantities may enter systemic circulation^7^. In experimental nanoparticle models, selected LNP formulations, particularly when specifically modified for brain targeting, can cross the blood-brain barrier (BBB) due to their small size, which allows them to evade the reticuloendothelial system, PEGylation for increased circulatory stability, and ligand functionalization enabling targeting of brain endothelial receptors, but the extent to which clinically used SARS-CoV-2 mRNA vaccine LNPs access the human brain remains uncertain ^8–10^.

A range of adverse events have been reported, likely related to the combined effects of the LNP-mRNA components and, in part, the biological activity of the S itself^7,8^. While mild side effects like fatigue and fever are common, rare severe neurological complications including acute disseminated encephalomyelitis (ADEM), autoimmune encephalitis (AE), and ischemic stroke have been reported in temporal association with vaccination^3,8,11–14^. According to the U.S. Vaccine Adverse Event Reporting System (VAERS), these events occur at a rate of approximately 0.03% of cases^10^. Despite their rarity and uncertain causality, the possibility of low-frequency neurological complications and uncertain underreporting rates raise concerns about LNP crossing the BBB and the pro-inflammatory effects of both the LNP-mRNA platform and the S^3,5–10^. However, to our knowledge, no prospective study has systematically investigated whether these vaccines cause brain changes detectable by MRI in healthy adults.

Conventional MRI techniques such as fluid-attenuated inversion recovery (FLAIR) improve the detection of brain lesions, including white matter hyperintensities (WMH), by improving contrast between pathological and normal brain tissue^2,3,12–17^. Quantitative T1 mapping (qT1) provides complementary information on tissue integrity and is sensitive to subtle microstructural changes, including blood-brain barrier disruptions, even in normal-appearing white matter (NAWM)^18–20^. High-resolution T1-weighted magnetization prepared rapid acquisition of gradient echoes (MP-RAGE) images derived from qT1 maps offer additional information on brain integrity^21^.

The hypothesis of this prospective pilot study was to exclude significant intraindividual changes in cerebral microstructure, including BBB integrity, evaluated through qT1, before and after mRNA-based LNP-modified SARS-CoV-2 vaccination of healthy adults. In addition, we systematically evaluated visually detectable macrostructural alterations using conventional 2D FLAIR and synthetic 3D MP-RAGE images.

## Methods

### Study design and participant selection

**Fig. 1:**
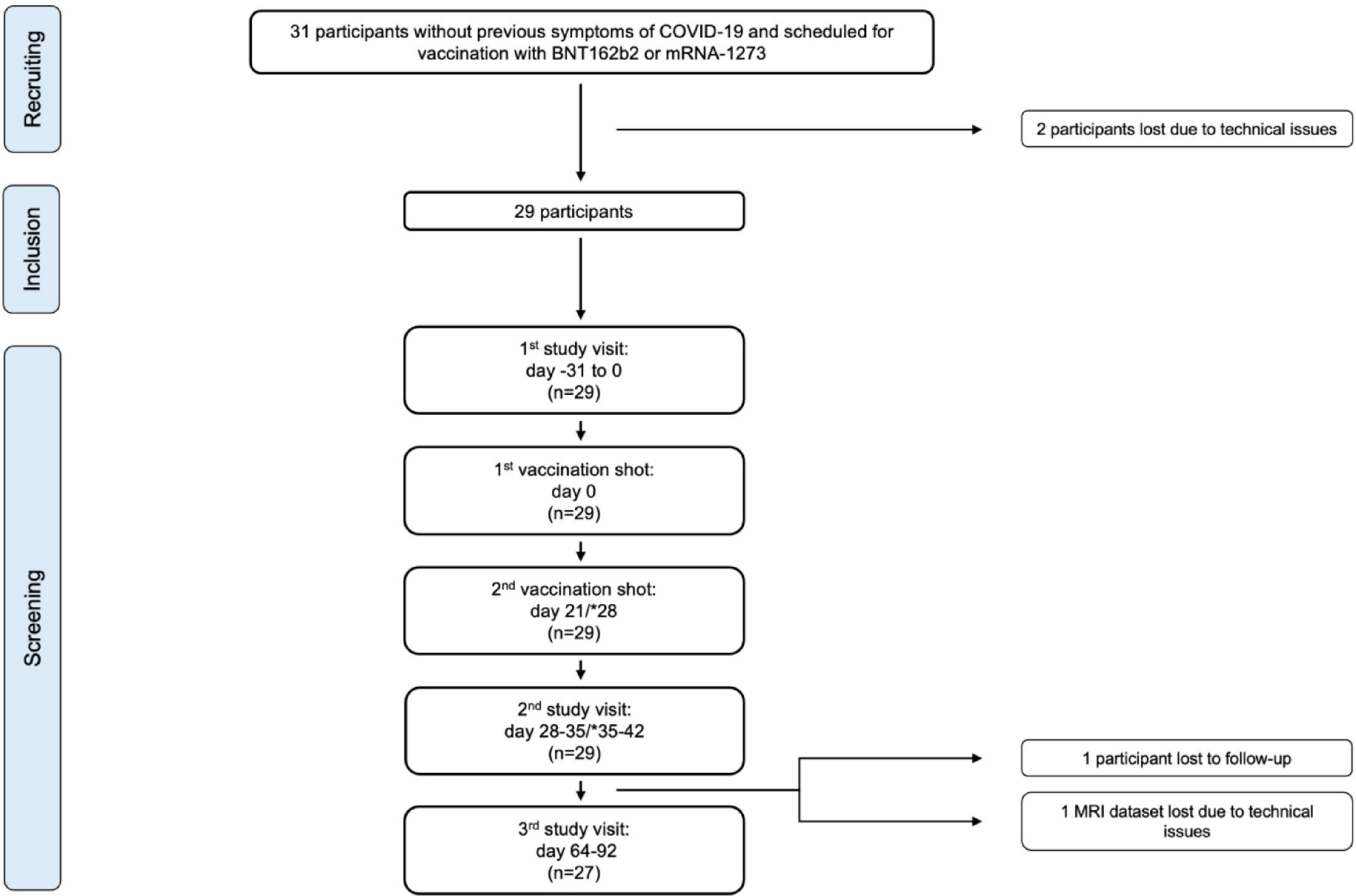
Recruiting, inclusion and screening. n = number of participants.

This prospective longitudinal study recruited healthy adult participants (aged ≥ 18 years) prior to their scheduled vaccination with BNT162b2 or mRNA-1273 at the University Hospital Frankfurt between February and May 2021. Participants underwent assessment at three time points: up to one month before their first vaccine dose as a baseline (t1), 1–2 weeks after the second dose (t2), and 2–3 months after the second dose (Fig. 1). Eligibility required absence of prior SARS-CoV-2 infection (confirmed by negative serological testing and self-report), absence of previous COVID-19 vaccination, chronic inflammatory or neuroinflammatory diseases, ongoing use of immunosuppressive drugs, pregnancy, or contraindication to MRI. Serological testing for antiviral immunoglobulin G and M (SARS-CoV-2 Rapid Antibody Test, Roche Diagnostics) and tympanic temperature measurement were performed at each time point.

The study was approved by the local ethics committee (ethics reference: 20–838) and funded by the Goethe Coronavirus Fund. It was conducted in accordance with the ethical guidelines for research involving human subjects, according to the Declaration of Helsinki. Participants gave informed consent prior to participation in the study.

### MRI acquisition and analysis

MRI datasets were acquired on a whole-body 3 Tesla scanner (MAGNETOM Prisma, Siemens Medical Solutions, Erlangen, Germany) with a body coil for radiofrequency transmission and a 20-channel phased-array head coil for radiofrequency reception. Detailed technical parameters of the MRI protocol are provided in Table 1. The protocol included a fat-saturated 2D axial FLAIR with an in-plane resolution of 0.8 × 0.8 mm² and a slice thickness of 3 mm, as well as 1 mm³ isotropic qT1 mapping, the latter using the variable flip angle method with FLASH-EPI hybrid readout for enhanced signal-to-noise ratio^22,23^. This approach involves acquiring two gradient echo datasets with distinct excitation angles, resulting in different varying degrees of T1- and proton density (PD)-weighting, from which qT1 values were derived based on contrast differences. Additional mapping of the radiofrequency field (B1) followed established protocols^24^. Static magnetic field (B0) distortions were corrected using B0 field maps matched to the geometry of the underlying qT1 acquisition, including voxel size, field of view, and spatial coverage. The correction accounted for local deviations of excitation angles that are induced by B0 distortions for water-selective excitation pulses, thus enhancing the accuracy of qT1 mapping which requires correct angle values. This method has been shown to improve qT1 measurements, particularly in basal brain regions, as described by Gracien et al.^25^.

**Table 1:** Acquisition parameters for FLAIR, qT1, B1 and B0 mapping, and synthetic MP-RAGE.

Protocol	FLAIR	qT1 (VFA-method)	B1 mapping	B0 mapping	MP-RAGE
Field of view (FOV) [mm]	210 × 210 × 149	256 × 224 × 160	256 × 224 × 160	256 × 224 × 160	256 × 224 × 160
TR [ms]	9000	16.4	11	560	1900
TE [ms]	81	6.7	5	4.89/7.35	0
TI [ms]	2500	-	-	-	900
Flip angle (α) [°]	150	4/24	11	60	9
Voxel size [mm]	0.8 × 0.8 × 3	1 × 1 × 1	4 × 4 × 4	4 × 4 × 4	1 × 1 × 1
Bandwith [Hz/pixel]	283	222	260	200	-
Acquisition time [min]	4:14	9:48	1:45	1:03	-

FLAIR = fluid-attenuated inversion recovery, MP-RAGE = magnetization prepared rapid acquisition of gradient echoes, qT1 = quantitative T1 mapping, TE = echo time, TI = inversion time, TR = repetition time, VFA = variable flip angle.

Computation of qT1 maps and data processing were performed with custom programs written in MATLAB (MathWorks, Natick, MA) and FSL (FMRIB, Software Library, v5.0, Oxford, UK, https://fsl.fmrib.ox.ac.uk/fsl)^26^. MP-RAGE-like anatomical datasets were synthetically generated from qT1 maps using methods outlined by Nöth et al.^21^. Briefly, pseudo proton density (PD) maps were derived from qT1 data as per Volz et al.^16^ and combined with quantitative relaxation information to generate T1-weighted contrast consistent with conventional MP-RAGE imaging^25^. This approach ensures intrinsic voxelwise correspondence between anatomical and quantitative data, thereby improving consistency for segmentation-based qT1 analyses. Importantly, the synthetic MP-RAGE data are derived from qT1 maps which have previously been corrected for inhomogeneities of the B1 field and receive profiles. Thus, they are free from any signal bias that is common in conventionally acquired data due to non-uniformities of the transmit and receive sensitivities of the radiofrequency coils used^25^. Synthetic MP-RAGE images served as the input for automated segmentation using FSL FIRST, including WM, cortical gray matter (GM), lateral ventricles, and key deep GM structures (thalamus, caudate nucleus, putamen, pallidum, amygdala, hippocampus and nucleus accumbens). These regions were selected due to their robust and reproducible segmentation properties and their established involvement in neuroinflammatory and infection-associated central nervous system (CNS) alterations^28–31^. This is particularly relevant in the context of inflammatory demyelinating conditions such as acute disseminated encephalomyelitis (ADEM), which have rarely been reported following SARS-CoV-2 infection or vaccination and may involve deep GM structures including the thalamus and basal ganglia in addition to subcortical WM lesions^14,32^. A partial volume threshold of > 80% was applied to minimize contamination from WMH, lacunes, and neighboring tissue compartments such as cerebrospinal fluid, while preserving sufficient voxel numbers for stable region of interest (ROI)-based qT1 estimation.

**Fig. 2:**
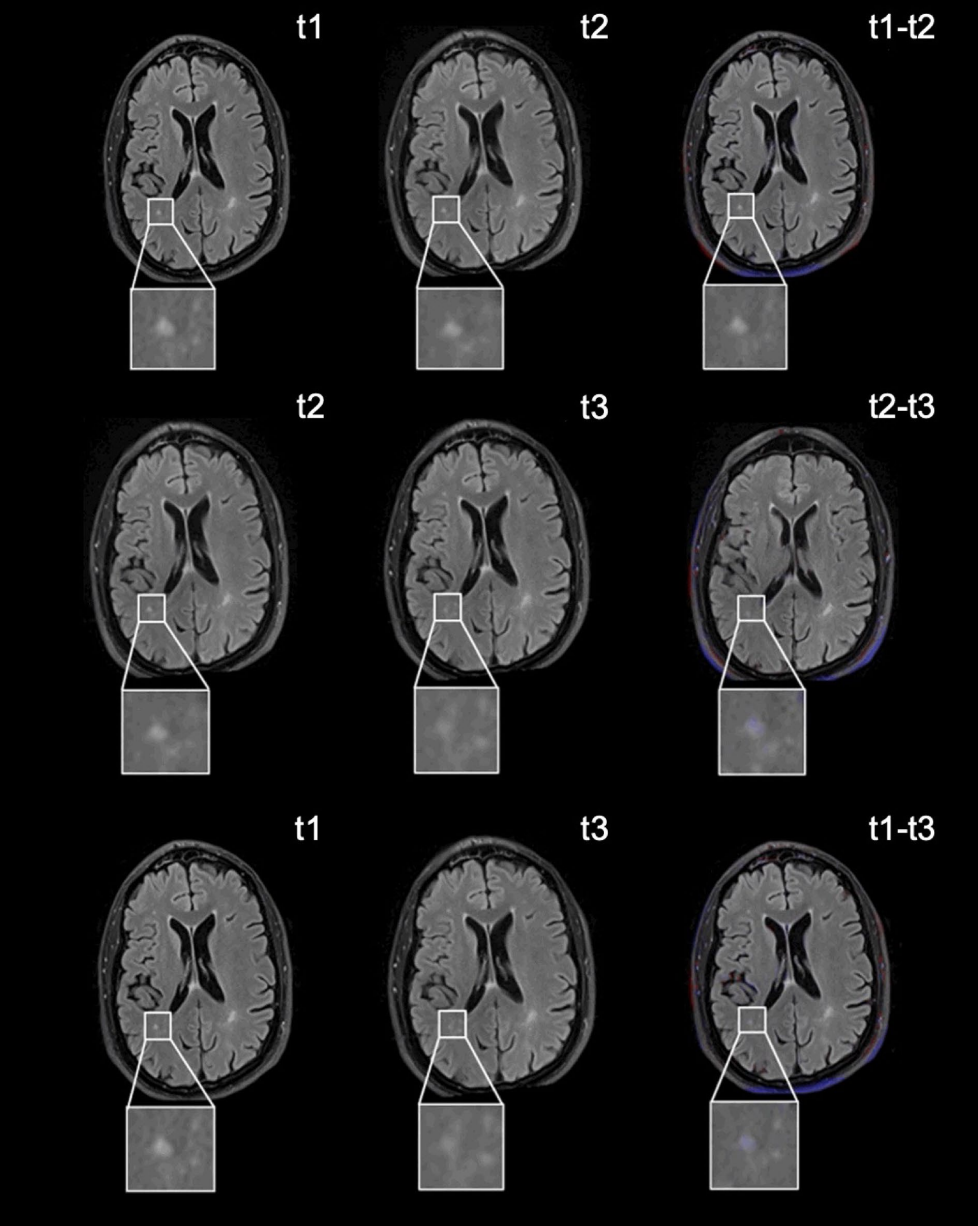
Semiquantitative analysis of white matter hyperintensity size between different time points. Analysis of fluid-attenuated inversion recovery (FLAIR) images using Longitudinal Brain Imaging (LoBI) application (IntelliSpace Portal, Philips Medical Systems, Best, The Netherlands). The blue color indicates a reduction in white matter lesion size, measured at 1 mm, located in the right parietal region, between timepoints 2 and 3 (t2-3) as well as between timepoints 1 and 3 (t1-t3).

3 mm FLAIR and 1 mm MP-RAGE images were visually assessed by one experienced neuroradiologist (C.T.A.) specialized in diagnostic neuroimaging (9 years of experience) for macrostructural brain abnormalities. WMH were defined as hyperintense lesions > 2 mm in size on FLAIR images and classified as either small vessel disease (SVD) or non-SVD, with SVD defined by the presence of bilateral, mostly symmetrical WMH in the deep WM^33^. The Prins scale^34^ was used to assess WMH change in number or size, assigning scores of -1 (decrease), 0 (no change) or 1 (increase). For semiquantitative analyses and detection of changes in WMH size on FLAIR images, and to confirm visual assessments, subtraction maps were generated between time points (t1 vs. t2, t2 vs. t3, and t1 vs. t3) using the Longitudinal Brain Imaging (LoBI) application (IntelliSpace Portal, Philips Medical Systems, Best, The Netherlands) (Fig. 2). For quantitative qT1 analyses, ITK-SNAP 3.6.0 was used to visualize, verify segmentation quality and extract qT1 values for each ROI across all time points.

### Statistical analysis

Statistical analyses were conducted using a custom script written in R (R Core Team, R: A Language and Environment for Statistical Computing, v4.3.0, Vienna, Austria, https://www.R-project.org/)^35^. The significance level was set at α = 0.05. Normality of distribution was assessed using the Shapiro-Wilk test. Continuous variables are presented as means with one standard deviation (SD) or medians with interquartile ranges (IQR), as appropriate. Paired t-tests or Wilcoxon signed-rank tests compared qT1 values in each ROI between time points. The Bonferroni correction was applied to adjust for multiple comparisons. In addition, the Benjamini-Hochberg procedure controlled for multiple testing and false discovery rate (with a 5% threshold). Fisher‘s Exact Test was applied to categorical variables.

To characterize longitudinal stability of qT1 values, intraindividual percent changes between timepoints were calculated for each ROI. For stability analysis, each participant was treated as a single statistical unit by averaging ROI-level percent changes across all timepoints, avoiding artificial inflation of the sample size. Based on published qT1 scan-rescan variability at 3 T with up to 2.14% (mean 1.23%)^36^, the a priori equivalence margin for physiologically acceptable variability was set to 2.14%. Equivalence analysis was performed by calculating the group-level mean percent deviation per subject with 95% confidence intervals. To quantify the study‘s sensitivity to detect deviations exceeding normal scan-rescan variability, a post hoc power analysis was performed using the observed intraindividual SD of percent changes. Power was calculated for detecting a deviation equal to the equivalence bound (2.14%) using a paired-differences design at α = 0.05. No inferential hypothesis testing was performed for stability analysis, as the objective was to evaluate observed variation relative to published scan-rescan limits rather than to test for statistical differences.

## Results

### Baseline characteristics

Detailed information on the recruited and excluded participants is described in Fig. 1. From February to May 2021, a total of 29 adults (13 males and 16 females; age range, 22 to 67 years; median age, 36 (17) years) (Table 2), were enrolled and underwent brain MRI at up to three time points, resulting in a dataset comprising 85 MRI scans. Twenty-nine participants completed imaging at t1 and t2, while 27 participants completed imaging at t3. One participant was lost to follow-up before t3, and one t3 examination could not be analyzed due to technical/server issues. Twenty-two participants received BNT162b2 and seven received mRNA-1273 (Table 2). All were tested seronegative for SARS-CoV-2 antibodies at initial MR examination and seropositive at both follow-up visits. No participants showed fever (> 37.9 °C) prior to any scan.

**Table 2:** Demographic data and classification of white matter hyperintensities.

Characteristics	*n*	%	Median	IQR
Sex				
Male	13	44.8		
Female	16	55.2		
Age			36	17
Vaccine				
BNT162b2	22	75.9		
mRNA-1273	7	24.1		
No WMH	10			
WMH	19			
SVD	7			
Non-SVD	12			

IQR = interquartile range, n = number of participants, SVD = small vessel disease, WMH = white matter hyperintensities.

### FLAIR and MP-RAGE image analysis

In the visual analysis, among the 29 participants, 10 showed no WMH and 19 exhibited WMH, of which 7 were classified as small vessel disease (SVD) and 12 as non-SVD (Table 2). Using the Prins scale, no participant showed any changes in WMH number or size across time points, as Fisher‘s Exact Test yielded *p* = 1 across all pairwise analyses. No new WMH suggestive of ADEM, post-ischemic territorial or lacunar defects, or other visually detectable macrostructural changes were observed longitudinally.

In the semiquantitative analysis using LoBI, one participant showed a 1 mm reduction in size of a single WMH between t2 and t3, and t1 and t3 (Fig. 2). No changes in lesion size or number were detected for any other participant.

### Quantitative T1 mapping analysis

Comparison of left and right hemispheric qT1 values of deep GM structures of interest (Table 3) showed a non-significant trend in the left hippocampus with a decrease from 1627 ± 61 ms (t1) to 1606 ± 59 ms (t3), corresponding to a -1.27% change (uncorrected *p* = 0.045). A similar trend was observed in the left accumbens nucleus with a decrease from 1643 ± 66 ms (t1) to 1611 ± 62 ms (t2), which equals a -2.08% change (uncorrected *p* = 0.040). Both changes did not remain significant after multiple comparison correction. Across all other deep GM structures of interest and whole cortical GM and WM, uncorrected p-values ranged from 0.194 to 0.915, and all remained non-significant after correction.

**Table 3:** Analysis of quantitative T1 relaxation times. Values are reported as mean ± standard deviation across subjects for each region of interest and time point.

Area		T1 value (ms) ± SD			Uncorrected p-value			Percent deviation (%)		
		t1	t2	t3	t1-2	t2-3	t1-3	t1-2	t2-3	t1-3
Deep GM	Left thalamus	1265 ± 41	1265 ± 45	1263 ± 35	0.940	0.846	0.852	0.07	-0.17	-0.08
	Right thalamus	1274 ± 42	1298 ± 38	1283 ± 35	0.598	0.822	0.123	1.80	-1.05	0.88
	Left caudate	1467 ± 42	1444 ± 50	1466 ± 33	0.157	0.111	0.895	-1.58	1.68	-0.07
	Right caudate	1429 ± 34	1425 ± 39	1424 ± 49	0.583	0.867	0.617	-0.32	-0.12	-0.35
	Left putamen	1339 ± 47	1334 ± 48	1336 ± 34	0.559	0.512	0.812	-0.43	0.37	-0.12
	Right putamen	1317 ± 48	1328 ± 41	1319 ± 46	0.268	0.379	0.675	0.66	-0.53	0.30
	Left pallidum	1071 ± 33	1062 ± 38	1059 ± 32	0.385	0.945	0.203	-0.80	-0.04	-0.97
	Right pallidum	1045 ± 36	1061 ± 31	1050 ± 32	0.082	0.191	0.370	1.39	-0.90	0.64
	Left hippocampus	1627 ± 61	1607 ± 55	1606 ± 59	0.100	0.979	**0.045**	-1.22	-0.02	-1.27
	Right hippocampus	1597 ± 44	1621 ± 65	1616 ± 57	0.081	0.628	0.066	1.34	-0.41	1.35
	Left amygdala	1635 ± 67	1615 ± 50	1611 ± 54	0.235	0.806	0.181	-1.31	-0.19	-1.34
	Right amygdala	1589 ± 69	1595 ± 61	1608 ± 65	0.864	0.340	0.238	0.17	0.98	1.27
	Left accumbens	1643 ± 66	1611 ± 62	1636 ± 59	**0.040**	0.155	0.669	-2.08	1.77	-0.33
	Right accumbens	1560 ± 60	1552 ± 59	1551 ± 72	0.173	0.891	0.583	-0.53	-0.13	-0.52
WM and cortical GM	Left WM	1113 ± 59	1118 ± 60	1108 ± 64	0.728	0.859	0.465	0.37	-0.52	-0.16
	Right WM	1103 ± 62	1117 ± 70	1106 ± 65	0.425	0.662	0.339	1.07	-0.65	0.53
	Left GM	1523 ± 29	1514 ± 36	1517 ± 25	0.236	0.915	0.194	-0.64	0.22	-0.37
	Right GM	1521 ± 30	1524 ± 23	1523 ± 29	0.508	0.721	0.576	0.19	-0.10	0.18

GM = gray matter, WM = white matter. Significant values are in bold (p < 0.05).

### Stability and post-hoc sensitivity analysis

Across all 85 MRI scans from 29 participants, intraindividual qT1 values showed small percent deviations between repeated measurements (mean 0.1%, SD 0.9%), symmetrically distributed, and centered around zero. The largest deviation was observed in the left nucleus accumbens (-2.08% between t1 and t2), corresponding to an absolute mean change of -32 ms. The group-level mean deviation per subject had a 95% confidence interval of -0.24% to + 0.44%.Post hoc sensitivity analysis indicated > 99% power to detect any deviation ≥ 2.14%. The individual-level minimal detectable change (MDC95) was 2.49%.

## Discussion

This pilot study aimed to investigate whether LNP-modified mRNA-based SARS-CoV-2 vaccines, specifically BNT162b2 and mRNA-1273, induce detectable macro- or microstructural brain changes, using conventional MRI and qT1 mapping. Our results support the hypothesis that these vaccines do not cause cerebral structural alterations in healthy adults.

Across 85 scans from 29 SARS-CoV-2-negative participants, we found no evidence of new or progressive WMH, nor any significant longitudinal intraindividual changes in qT1 values across brain regions. Visual analysis revealed no significant macrostructural changes in size or number of WMH following vaccination. Semiquantitative analysis using LoBI identified only a small reduction of 1 mm in the size of a single WMH in one participant between t2 and t3, and between t1 and t3. However, this lesion was already present prior to the first vaccination and small size reductions can also occur in the context of SVD^37^.

While conventional MRI misses subtle changes in brain microstructure including the BBB integrity, qT1 is sensitive to such changes^18–20,25^. Statistical analysis of qT1 values as quantitative parameters for deep GM structures and whole cortical GM and WM in both hemispheres revealed no evidence of microstructural changes after applying the Bonferroni and Benjamini-Hochberg corrections, supporting the robustness of our findings.

QT1 values showed high longitudinal stability (mean 0.1%, SD 0.9%). Notably, minor uncorrected decreases in T1 values of the left hippocampus (-21 ms, -1.27%, *p* = 0.045) between t1 and t3, and left accumbens nucleus (-32 ms, -2.08%, *p* = 0.040) between t1 and t2 were observed. A corresponding trend was present in the contralateral hippocampus (19 ms, 1.35%, *p* = 0.066). While the hippocampus is a known predilection site for AE, both the hippocampus and accumbens nucleus are functionally linked to the limbic system, which has led to speculation that these regions may be vulnerable to inflammatory alterations in SARS-CoV-2-related conditions^1–3,28,38,39^. In this regard, similar qT1 changes in hippocampal and striatal regions have been reported following moderate-to-severe SARS-CoV-2 infection, supporting the notion that these structures may be sensitive to systemic inflammatory or infectious processes^28^. However, the present cohort differs fundamentally in exposure, disease severity, and inflammatory burden, and no comparable pattern of regional or hemispheric involvement was observed. Importantly, all observed changes remained within established scan-rescan variability limits for qT1 at 3 T and showed no spatial or temporal consistency across regions or time points^36^. Equivalence analysis further supported the stability of qT1 values: the 95% confidence interval of the group-level mean deviation (-0.24% to 0.44%) was entirely contained within the predefined variability margin of 2.14%^36^. Post hoc sensitivity analysis indicated > 99% power to detect deviations exceeding 2.14%, and the individual minimal detectable change (MDC95 = 2.49%) exceeded all observed changes. Together, our data suggest high stability of neuroimaging parameters following vaccination. Nonetheless, this does not fully exclude the possibility of transient or rare effects in susceptible adults.

Neurological complications such as ADEM and AE, characterized by hyperintense lesions on FLAIR images^2,3,13–18^, have been reported after both SARS-CoV-2 mRNA vaccinations^3,8,11–14^ and SARS-CoV-2 infections^1–3,12^, highlighting the complexity of distinguishing between vaccine-related and virus-induced neurological conditions.

Demyelinating diseases such as ADEM have been investigated using quantitative imaging techniques like apparent diffusion coefficient (ADC), diffusion tensor imaging (DTI) and magnetic resonance spectroscopy (MRS) to assess changes in NAWM^16,17^. QT1 has proven sensitive in detecting subtle microstuctural abnormalities in NAWM in diseases like multiple sclerosis^20^. Building on this concept, we specifically applied qT1 mapping to assess potential microstructural alterations in NAWM of our post-vaccination cohort, as this technique enables sensitive, quantitative detection of early tissue changes that may not be visible on conventional MRI. To our knowledge, no qT1 analyses of NAWM in patients with potentially subtle inflammation after vaccination (i.e., ADEM) have been conducted yet.

Symptoms typically manifest a median of 14 days following SARS-CoV-2 vaccination, with a higher frequency after the first dose, and cases reported more than 2 months after vaccination are rare^3,14^. Our imaging schedule included two post-vaccination scans after the second dose but did not contain an early scan after the first dose. Since our 2-3-month follow up exceeds the typical temporal window of expected vaccine-related demyelinating pathologies like ADEM, it is unlikely that ADEM occurred after t3. However, we cannot fully exclude short-lived signal changes occurring during the early window between t1 and t2 as well as between t2 and t3, although this is unlikely, as ADEM lesions generally resolve within 3 to 24 months, with a median resolution time of 7.2 years^40^.

Patients with neurological complications and MRI findings indicative of active demyelination following BNTb162b2 or mRNA-1273 vaccines have shown favorable responses to corticosteroids, plasma exchange and intravenous immunoglobulin with most recovering significantly or completely, while the others achieved stabilization. Importantly, studies have shown that the risk of demyelinating lesions is substantially higher after SARS-CoV-2 infection than after vaccination, underscoring the protective benefit of vaccination^12,14^.

Despite experimental evidence suggesting potential pro-inflammatory effects of the S and LNP-mRNA crossing the BBB, our data revealed no detectable structural changes within the sensitivity of the applied MRI methods^3,5–10^. Notably, LNP are intentionally used to cross the BBB in therapies such as chemotherapy, immunotherapy, gene therapy, and combination therapy for glioblastoma, highlighting their potential for therapeutic applications^9^.

It is important to consider our study‘s results in light of its limitations. First, the monocentric sample of 29 participants restricts generalizability and statistical power to detect rare post-vaccination effects. However, this exploratory pilot study was designed to assess prospective intraindividual subtle microstructural alterations using qT1 mapping rather than estimate event prevalence. Larger multicenter studies are therefore needed to confirm the broader relevance of these findings. Second, although 2D FLAIR imaging was used in accordance with established demyelination protocols, isotropic 3D FLAIR sequences might improve sensitivity for subtle lesion changes in future studies. Further, no dedicated volumetric ROI-based analysis (e.g., hippocampal or whole-brain volumetry) was performed, as substantial macrostructural changes were not expected within the relatively short follow-up period and exploratory sample size. Moreover, extensive volumetric analyses would have increased susceptibility to segmentation variability and multiple-testing bias. Therefore, the analysis focused on longitudinal within-subject assessment of quantitative MRI parameters and visually detectable structural changes. Nonetheless, subtle macrostructural alterations below the detection threshold of visual inspection cannot be fully excluded. Furthermore, the pseudo PD maps used for synthetic MP-RAGE generation represent an approximation of directly measured PD maps and may introduce estimation bias in some regions^25^. Variability measures reflect between-subject variability at the ROI level, as voxel-wise intra-ROI distributions were not retained in the exported dataset. Additional limitations include the lack of voxel-wise analysis, restriction of ROI assessments to major regions and omission of the brainstem. The use of synthetic MP-RAGE rather than independently acquired T1-weighted imaging represents a deliberate methodological choice within a fully quantitative MRI framework. This approach ensures intrinsic voxel-wise correspondence between anatomical segmentation and qT1 parameter maps, thereby improving consistency of ROI-based analyses in longitudinal settings. In addition, it reduces potential variability introduced by separate structural acquisitions, including differences in receiver coil sensitivity, intensity inhomogeneities, and sequence-specific distortions.

Although data acquisition was completed in early 2021, additional time was required for longitudinal quality control, validation of the quantitative MRI processing workflow, and optimization of synthetic image generation before final statistical evaluation and manuscript preparation. Furthermore, manuscript completion and submission were delayed by extensive clinical and academic responsibilities during and after COVID-19 pandemic.

In summary, our data do not support the presence of short-term microstructural brain alterations following mRNA-based SARS-CoV-2 vaccination in healthy adults. Neither conventional MRI sequences nor qT1 as advanced technique revealed structural abnormalities including signs of BBB dysfunction. QT1 values remained highly stable over time. Although this study was not designed to detect extremely rare complications, the absence of MRI-detectable changes in our dense dataset adds to the growing evidence supporting the neurological safety of mRNA vaccination.

## Data Availability

Data generated and/or analyzed during the study are available from the corresponding author on reasonable request.

## Abbreviations

ADEM: Acute disseminated encephalomyelitis
BBB: Blood-brain barrier
COVID-19: Coronavirus disease 2019
FLAIR: Fluid-attenuated inversion recovery
GM: Gray matter
LNP: Lipid nanoparticles
MP-RAGE: Magnetization prepared rapid acquisition of gradient echoes
mRNA: Messenger ribonucleic acid
PD: Proton density
ROI: Region of interest
S: Spike glycoprotein
SARS-CoV-2: Severe acute respiratory syndrome coronavirus 2
SVD: Small vessel disease
WM(H): White matter (hyperintensities)
